# Experiences and impact of living with incontinence associated stigma: A protocol for a systematic review and narrative synthesis of qualitative studies

**DOI:** 10.1371/journal.pone.0270885

**Published:** 2022-07-08

**Authors:** Cathy Murphy, Miriam Avery, Margaret Macaulay, Mandy Fader

**Affiliations:** University of Southampton, Southampton, United Kingdom; University of Ottawa, CANADA

## Abstract

**Introduction:**

Incontinence is global health and social issue, with urinary incontinence alone affecting over 400 million people. Incontinence can lead to physical harms such as skin damage, but it also commonly causes social and psychological harms, including those associated with stigma. For many people, treatment to cure incontinence does not work or is not suitable and they live with the long-term consequences of incontinence. At the moment, no stigma reduction interventions (increasingly used with other conditions such as mental health problems and HIV) have been developed for people living with incontinence. As a starting point for developing such an intervention, this review will address the questions 1) What are the incontinence (urinary or faecal) associated experiences of stigma of people living with incontinence? 2) What is the impact of incontinence associated stigma on their lives?

**Methods:**

The reviewers will search Embase, Medline, PsychINFO and the Cumulative Index to Nursing and Allied Health Literature using controlled vocabulary and relevant search terms. Articles assessed to meet inclusion criteria will be included. Once duplicates have been removed, titles and abstracts will be screened and full texts of selected research articles will be reviewed. An adapted Joanna Briggs Institute Data Extraction Form will be used to collect the data and quality will be assessed using the Joanna Briggs Institute checklist for qualitative research appraisal tool. A framework approach (using the Revised Framework for Understanding Non-communicable Disease Related Stigma) will be used to organise, integrate, interpret and summarise findings from included articles. The review will be reported in accordance with the Enhancing Transparency in reporting the synthesis of qualitative research statement. Prospero registration number CRD42021259065.

**Discussion:**

The systematic review described in this protocol will provide the first in-depth, comprehensive understanding of people’s experiences of the stigma associated with incontinence and the impact that it has on their lives. It will identify broader influences of contextual variables such as age, sex, cause and type of incontinence, socio-economic culture and geographical location. The review aims to provide insights to support the development of incontinence associated stigma reduction interventions.

## Introduction

Incontinence (the involuntary leakage of either urine or faeces) is recognised as a significant global health issue [1–3]. Urinary incontinence alone is estimated to affect 12% of women and 5% of men worldwide [4]. Incontinence (either urinary or faecal) is a symptom with many underlying causes. Many major conditions such as some cancers [5, 6], stroke [7], obesity [8], dementia [9], inflammatory bowel disease [10], childbirth [11] and menopause [12] are linked to an increased risk of incontinence.

Healthcare professionals should always seek to reverse the cause of incontinence, but this is often not possible or can take time; many people live with temporary or permanent incontinence [13]. There is a myriad of physical and psychological harms associated with incontinence, including skin breakdown [14], increased risk of falls [15], depression and anxiety [16]. One commonly reported incontinence associated harm is the experience of stigma and the negative impact on people’s quality of life is widely acknowledged [17].

Goffman’s 1963 seminal work explained that stigma is directed at people perceived to have an undesirable attribute that sets them apart from others by others in society [18]. Stigma is now well-documented as a driver of poor health outcomes [19]. Numerous frameworks and models building on Goffman’s work have been developed, including Bos et al’s conceptualisation of four interrelated manifestations of stigma; public stigma (reactions to someone perceived to have a stigmatising condition), self-stigma (impact of possessing a stigma), stigma by association (reactions to people associated with a stigmatised person) and structural stigma (perpetuation of stigmatised status by institutions and ideology) [20]. The impact of stigma can be difficult to estimate, but it is known to affect important outcomes such as help seeking, quality of life and care engagement [21–23]. Therefore, it is unsurprising that a considerable amount of work has been put into developing stigma reduction interventions (SRI) for other high stigma conditions such as epilepsy [24], HIV/AIDS [25] or mental health disorders [26]. However, there are no reports of the development or use of SRIs for incontinence associated stigma. This is despite the fact that incontinence is considered significantly more taboo than, for example, cancer or depression [27].

A rapid review of the literature on incontinence associated stigma undertaken to support designing this protocol demonstrated that, whilst it is highly prevalent and harmful, stigma and its impact appears to vary with group characteristics, for example, the underlying cause of incontinence [28], sex of the person [29], cultural differences [30] and type and severity of incontinence [31, 32] all seem to play a role in the experience of stigma. However, as yet, no systematic review on the topic has been published and understanding is limited. It is hoped that the review described in this protocol will provide greater understanding of incontinence associated stigma and consequently aid the development of one or more SRIs with the potential to decrease the negative impact of stigma.

Given the knowledge gaps in this area, the proposed systematic review with narrative synthesis of qualitative research aims to address the questions:

1. What are the incontinence associated experiences of stigma of people living with incontinence?
2. What is the impact of incontinence associated stigma on people’s lives?

The objective of the review is to identify, appraise and synthesise findings from qualitative studies (including qualitative elements of mixed method studies) that address the research questions. The review will include existing evidence provided by people living with incontinence and also their unpaid carers. It is important to include the views of unpaid carers for two reasons; firstly to avoid the exclusion of populations who might otherwise have a less heard voice (e.g. those with dementia or learning difficulties) and secondly because the stigma of incontinence has an impact on carers and the caring relationship that warrants investigation.

## Methods

### Study design

A systematic review of published evidence on the experiences and impact of incontinence associated stigma will identify and synthesise what is known on the topic, providing a comprehensive and structured understanding of qualitative research in the area. Using a framework-based qualitative synthesis approach, the review will appraise and interrogate results from included papers with the aim of providing insights into the experiences and impact of incontinence associated stigma. The processes described will be undertaken by at least two members of the team, with any differences discussed by at least three team members.

The framework approach to thematic synthesis will be facilitated by the use of Rai and colleagues’ Revised Framework for Understanding Non-communicable Disease Related Stigma [32]. This framework provides a conceptualisation of stigma in five categories: 1) sources of stigma,

2) biopsychosocial mechanisms/drivers (causative mechanisms), 3) manifestations of stigma, 4) consequences of stigma and 5) mitigating factors/strategies to curb/overcome stigma. This framework was chosen as it provides both sufficient conceptual breadth to include a range of non-communicable conditions (such as incontinence) and a useful organisational structure for findings from different populations. Use of this framework is described in the Data Extraction and Synthesis section.

### Protocol registering and reporting

The review will be reported in accordance with the Enhancing Transparency in Reporting the Synthesis of Qualitative Research (ENTREQ) [33] statement, as was this protocol. Additionally, the Preferred Reporting Items for Systematic Reviews and Meta-Analyses Protocols (PRISMA-P) [34] statement was used to guide reporting in this protocol. This review protocol has been registered with the International Prospective Register of Systematic Reviews (PROSPERO) database (registration number CRD42021259065).

### Eligibility criteria

The SPIDER (Sample, Phenomenon of Interest, Design, Evaluation, Research type) tool [35] was used to support the development of both the research question and eligibility criteria. The inclusion criteria are as follows.

- Sample: People (from any clinical population or their unpaid carers) living with urinary and/or faecal incontinence who describe the experience and/or impact of stigma associated with the incontinence.
- Phenomenon of interest: Incontinence associated stigma.
- Design: Any qualitative research design (e.g. interviews or ethnography), including qualitative results from mixed-methods studies.
- Evaluation: Descriptions of the experience or impact of incontinence associated stigma.
- Research Type: Primary qualitative research.

The exclusion criteria are:

1. Studies that do not meet the inclusion criteria.
2. Studies not published in English.
3. Studies that do not include a qualitative element.
4. Studies reporting the perspectives of people who are not the person living with incontinence or the unpaid carer of a person living with incontinence (e.g. healthcare professionals).
5. Studies that include brief reference to stigma in passing rather than a major theme or topic.

### Search strategy

The key information source will be a structured search of literature in electronic databases: Embase, PsychINFO, MEDLINE and CINAHL. Literature from inception onwards will be searched. A title only search will be undertaken on Google Scholar using guidance from Haddaway et al [36], with the first 1000 results screened for eligibility. Additionally, Proquest Dissertations & Theses Global will be searched. The reference lists of included articles will be hand-searched for potentially eligible studies. The list of search terms will be devised and finalised in collaboration with a health sciences librarian using a combination of controlled vocabulary and key terms for the concepts in the inclusion criteria.

### Study selection and data management

Following the removal of all duplicates, the database search and hand-searching will be performed with all titles and abstracts screened to remove studies that are clearly not relevant, guided by the eligibility criteria. The full text of remaining studies will be retrieved and reviewed. Where there is any question on study eligibility, the review team will discuss the study to reach a decision. A reason for exclusion will be recorded on the PRISMA flow-chart for each non-eligible study. The articles assessed to meet the inclusion criteria will be retained and included in the synthesis. Articles will be managed using EndNote Web, with new databases used to manage each stage of the process.

### Data extraction and synthesis approach

Using a custom designed Microsoft Excel form (adapted from the Joanna Briggs Institute data extraction form), data will be extracted from each article on author, year of publication, geographic location, method, setting, population (e.g. clinical group), participant demographics, relevant results and comments. All data describing experiences of stigma or the impact of stigma will be extracted, including verbatim participant quotes. The results section of the data extraction form will be developed to capture data using the domains of the Framework for Understanding Non-communicable Disease related stigma [29]. Synthesis of the data will be undertaken by tabulating individual study data by domain. Variation between different groups (e.g. by sex or incontinence type) will be actively sought by the reviewers and developed into sub-domains where appropriate. Data will be explored within and across domains (and sub-domains) and studies, and grouped by relevant characteristics, for example underlying condition (e.g. prostate cancer or fistula), age or sex where sufficient data allows.

### Quality appraisal

All eligible articles will be appraised for their quality using the Joanna Briggs Institute’s Critical appraisal Checklist for Qualitative Research assessment tool [37] and the results summarised. This tool has been used extensively and judged to provide a coherent assessment [37]. The appraisal process will help to determine whether included studies meet accepted quality standards and identify any limitations.

### Confidence in cumulative evidence

The GRADE-CERQual (“Confidence in the Evidence from Reviews of Qualitative research”) approach will be used to report the level of confidence in whether the review findings provide a reasonable representation of the phenomenon of interest [38]. The four areas of assessment are: 1) methodological limitations, 2) coherence, 3) adequacy of data and 4) relevance [38]. The ‘Applying GRADE-CERQual to qualitative evidence synthesis findings’ set of papers will be used to guide the process [39–42]. Any ambiguities will be discussed by the review team in order to reach a decision.

### Ethics

Ethical review will not be sought for conducting this systematic review as individual level data will not be accessed.

## Discussion

This systematic review will provide for the first time a comprehensive and nuanced understanding of people’s experiences of incontinence-associated stigma and the impact that it has on their lives (and those of their unpaid carers). It will identify broader influences of contextual variables such as age, sex, underlying cause and type of incontinence, socio-economic culture and geographical location. By synthesising literature from a range of populations, we will be able to identify similarities and variations between groups that will support a thorough and cohesive characterisation of the phenomenon.

It is expected that this work will support clinicians, policy-makers and researchers who are trying to improve the quality of life of people living with incontinence and their carers. In particular, it will aid the development of one or more Stigma Reduction Interventions for people living with incontinence. Better understanding the phenomenon of incontinence associated stigma, will improve the chances of developing a targeted and effective SRI. Additionally, it is likely that this review will contribute new knowledge to support existing conceptual frameworks for understanding the experience of living with and caring for incontinence, for example the Dignity in Continence Care Framework [43]. Such frameworks provide a holistic understanding of incontinence and caring for incontinence, thus playing an important role in attempts to improve care.

There are some potential limitations to this study. As with all reviews of qualitative research, the findings of this review will be dependent on the methodological quality and reporting standards of the original studies. It has been observed that quality appraisal of qualitative studies can be problematic, in part due to the lack of agreement between different communities of researchers on how the value of such work should be assessed [44]. The use of the Joanna Briggs Institute’s Critical appraisal Checklist for Qualitative Research assessment tool [37] will go some way to mitigating these issues. An additional potential limitation is the variation in the definition of stigma used in the studies. The concept of stigma has been criticised for being too vague [45] and lacking a theoretical perspective [46]. The use of Rai and colleagues’ [32] framework and the review’s broad definition of stigma will help to address this concern.

## Supporting information

S1 Checklist PRISMA-P 2015 checklist. (DOCX)
